# Regional drivers of clutch loss reveal important trade-offs for beach-nesting birds

**DOI:** 10.7717/peerj.2460

**Published:** 2016-09-13

**Authors:** Brooke Maslo, Thomas A. Schlacher, Michael A. Weston, Chantal M. Huijbers, Chris Anderson, Ben L. Gilby, Andrew D. Olds, Rod M. Connolly, David S. Schoeman

**Affiliations:** 1 Ecology, Evolution and Natural Resources, Rutgers, The State University of New Jersey, New Brunswick, NJ, United States; 2 School of Science and Engineering, University of the Sunshine Coast, Maroochydore, Australia; 3 Centre for Integrative Ecology, Deakin University, Geelong, Australia; 4 Australian Rivers Institute - Coast & Estuaries, and School of Environment, Griffith University, Gold Coast, Queensland, Australia

**Keywords:** Shorebirds, Sandy shore, Egg loss, Predators, Flood, Seascape

## Abstract

Coastal birds are critical ecosystem constituents on sandy shores, yet are threatened by depressed reproductive success resulting from direct and indirect anthropogenic and natural pressures. Few studies examine clutch fate across the wide range of environments experienced by birds; instead, most focus at the small site scale. We examine survival of model shorebird clutches as an index of true clutch survival at a regional scale (∼200 km), encompassing a variety of geomorphologies, predator communities, and human use regimes in southeast Queensland, Australia. Of the 132 model nests deployed and monitored with cameras, 45 (34%) survived the experimental exposure period. Thirty-five (27%) were lost to flooding, 32 (24%) were depredated, nine (7%) buried by sand, seven (5%) destroyed by people, three (2%) failed by unknown causes, and one (1%) was destroyed by a dog. Clutch fate differed substantially among regions, particularly with respect to losses from flooding and predation. ‘Topographic’ exposure was the main driver of mortality of nests placed close to the drift line near the base of dunes, which were lost to waves (particularly during storms) and to a lesser extent depredation. Predators determined the fate of clutches not lost to waves, with the depredation probability largely influenced by region. Depredation probability declined as nests were backed by higher dunes and were placed closer to vegetation. This study emphasizes the scale at which clutch fate and survival varies within a regional context, the prominence of corvids as egg predators, the significant role of flooding as a source of nest loss, and the multiple trade-offs faced by beach-nesting birds and those that manage them.

Several iconic, threatened species of the world’s coastlines nest on ocean-exposed sandy shores (e.g., turtles, birds) and are thought to use nest-site selection to increase clutch success, hatchling survival, and ultimately fitness (Refsnider & Janzen, 2010; Spencer, 2002). Sandy shores include distinct habitat types (dunes, non-vegetated beach, surf-zone), each with variable attributes and subject to a variety of pressures (e.g., predators, people, urban development) (Meager, Schlacher & Nielsen, 2012; Schlacher, Meager & Nielsen, 2014). Heterogeneity of the sandy beach environment across space and time implies that reproductive success for these species might depend on their flexibility in finding suitable nest sites.

Coastal birds are important contributors to sandy beach and dune ecosystems, transferring resources between marine and terrestrial systems (Huijbers et al., 2015), providing critical nutrient inputs (Sekercioglu, 2006), and sometimes filling the role of apex consumers (Brown et al., 2015). They also act as surrogates for beach and dune conservation and may be prime indicators of beach condition (Schlacher, Meager & Nielsen, 2014). Despite their significant role in ecosystem function and management, persistence of many coastal bird populations is severely threatened by direct and indirect anthropogenic impacts (e.g., Brinker et al., 2007; Dowding & Murphy, 2001; Van De Pol et al., 2010). Viability of resident coastal bird populations is limited by failed nesting attempts (i.e., clutch failure) and high chick mortality resulting from predation, flooding, and human disturbance (Erwin et al., 2006; Martín et al., 2015; Tjørve & Underhill, 2008).

For decades, researchers have examined potential factors influencing clutch survival of coastal birds. They have identified a suite of egg and chick predators across several taxonomic groups (Brooks et al., 2014; Ivan & Murphy, 2005), evaluated links between direct and indirect human interference and reproductive failure (Ruhlen et al., 2003; Weston & Elgar, 2007), and quantified the impact of high tides and storms on reproductive success (Brooks et al., 2013; Pol et al., 2010). These studies were generally performed on small sections of coastline under relatively uniform management and human use regimes (e.g., Dutton et al., 2005; Neuman et al., 2004), and were assumed to experience similar conditions (e.g., Hardy & Colwell, 2012; Morse, Powell & Tetreau, 2006). Thus, we have a good understanding of threats coastal ground-nesting birds encounter at local scales. For example, nests on beaches that are used heavily for human recreation may experience higher direct mortality from trampling, crushing, and vandalism (Brooks et al., 2013; Pol et al., 2010); these areas might also support higher predator densities due to the provision of additional food resources from garbage (Yasué & Dearden, 2006). Nests on uninhabited beaches may experience less chronic human disturbance, but be more vulnerable to severe acute human impacts due to a lack of regulatory presence and enforcement (Brown et al., 2015; Del Viejo et al., 2004). The suite of predators may also vary in response to variations in landscape connectivity, habitat types and the presence of invasive species (Brown et al., 2015). While such studies are important in understanding local threats and informing local management, it remains unclear whether outcomes are more broadly applicable.

Conservation practitioners have at their disposal a portfolio of management options that can be applied on a site-by-site basis (Maslo & Lockwood, 2009; Neuman et al., 2004). Management at a given site can improve reproductive success of a target species, although it can be costly (e.g., Hecht & Melvin, 2009). Further, managers must continuously monitor changing site conditions to determine when and how to appropriately intervene (Cohen et al., in press). Mitigation of all threats places a heavy burden on beach-nesting bird managers, who are already constrained for time and resources, and such efforts may be fruitless if nests are lost to flooding. Importantly, even the most effective management at the local scale does not guarantee population viability. Understanding the pressures reducing clutch success at a regional scale may relieve some of the management burden by allowing managers to prioritize threat mitigation within or across regions. Identifying the relative importance of the generalized predictors of clutch failure across a regional scale may also increase population-level benefits of management. Managers would increase both their confidence in deciding appropriately where and when to apply specific interventions, as well as the efficiency with which management is implemented.

Determination of clutch fate of beach-nesting birds historically has been elusive (Ivan & Murphy, 2005; Mabee, 1997), preventing the identification of generalized predictors of clutch failure. Few studies investigate clutch survival over spatial scales that are large enough to encompass different environmental conditions or gradients in anthropogenic pressures. To address this gap, we examined beach-nesting bird clutch fate (using a model system of shorebird nests and eggs) along 180 km of coastline selected to encompass a diversity of geomorphological, anthropogenic, and predator environments. We identified the primary causes of clutch loss among a suite of putative factors at a regional scale. By definitively assigning clutch fate to a set of artificial nests distributed across varying beach habitats and examining an exhaustive list of predictor variables hypothesized to influence clutch failure, we determined the relative importance of predation, flooding, and human disturbance on the probability of clutch loss.

## Study Area

**Figure 1:**
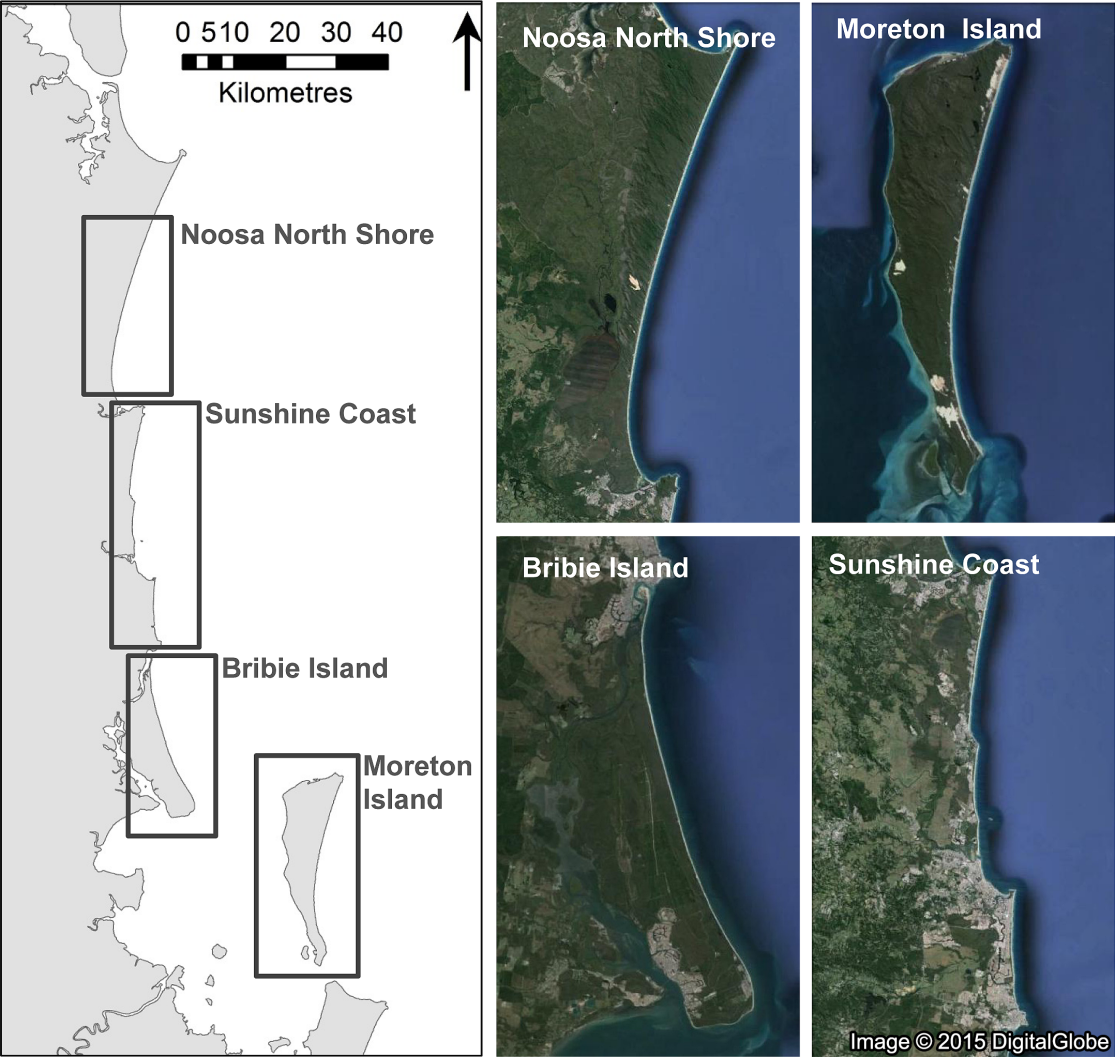
Location of study regions in southeast Queensland in eastern Australia where we monitored experimental nests on beaches and dunes on Moreton Island (*n* = 38), Bribie Island (*n* = 27), the Noosa North Shore (*n* = 38), and the Sunshine Coast (*n* = 30) in early 2015. Image: Google, 2015 Digital Globe.

We purposefully selected four study sites within the region of southeast Queensland, Australia that differed in the types of likely mortality agents present, including two sand-barrier islands (Bribie and Moreton Islands) and two mainland sections of the ocean-exposed coastline (Noosa North Shore and Sunshine Coast; Fig. 1). Sites were separated between 12 and 27 km. The main differences between sites were the presence of exotic and feral predators (red foxes, *Vulpes vulpes*; dogs, *Canis familiaris*); the intensity of development and human use; and the types of recreational activities (camping, fishing, off-road vehicles; Table 1). Basic habitat attributes were similar between sites, all being ocean-exposed sandy beaches of intermediate morphodynamic state, backed by generally low (typically 2–4 m) foredunes of 10–40 m width (Brown et al., 2015; Huijbers et al., 2015; Huijbers et al., 2013; Schlacher & Thompson, 2012; Schlacher & Thompson, 2013a; Schlacher & Thompson, 2013b; Schlacher et al., 2015a). Local geomorphology varied, however, along these coasts (e.g., width and steepness of dunes and beaches), so the study area also provided an opportunity to examine the influence of these variables on clutch survival.

**Table 1:** Comparison of study regions within SE-Queensland, Australia with respect to likely causes of clutch mortality associated with shore- and dune-nesting birds. Assessments are based on five years of field studies at these locations by TAS and MW.

Region	Foxes, Dogs, Cats	Off-road vehicles	Dune camping	Urban Development
Moreton Island	no	yes	yes	none
Bribie Island	yes	yes	limited	moderate
Noosa North Shore	yes	yes	yes	sparse
Sunshine Coast	yes	no	no	intense

## Methods

We experimentally mimicked nests of red-capped plovers, *Charadrius ruficapillus*, and monitored with camera traps the fate of clutches (after Cardilini et al., 2013). Red-capped plovers are widely distributed across Australia and breed in Queensland throughout the year, with a peak in spring and summer (Marchant & Higgins, 1993). Females typically lay two eggs of cryptic colouration in a shallow scrape in the sand. For beach-nesting individuals, most nests are located on the upper beach and in foredunes, either in the open, under vegetative cover, or next to flotsam objects (Ekanayake et al., 2015a; Marchant & Higgins, 1993).

To mimic plover clutches, we placed two European quail (*Coturnix japonica*) eggs in shallow sand scrapes; quail eggs are approximately the same size, weight and colour as red-capped plover eggs and have been used previously for these types of experiments (Maguire, Stojanovic & Weston, 2010). We used survival of the model clutches as an index of real clutch survival, whilst acknowledging that the presence and behaviour of an incubating adult may alter absolute clutch loss rates (Smith, Gilchrist & Smith, 2007). However, there is no a priori expectation that systematic bias amongst regions would result from using experimental (model) clutches. We obtained unwashed quail eggs from a local hatchery 1–2 days prior to field deployment and handled them as little as possible. The quail eggs used in this experiment were 32.5 ± 0.13 (SE) mm long and 24.8 ± 0.07 mm wide, and weighed 10.8 ± 0.10 g (real Red-capped Plover eggs are 30.42 ± 0.15 mm long and 22.42 ± 0.07 mm wide; *n* = 73 MA Weston, 2016, unpublished data).

### Model clutch survival experiments

We ran experiments during the late summer and early autumn of 2015 (Moreton Island: 31 Jan–07 Feb; Noosa North Shore: 3–9 March; Bribie Island: 13–19 March; Sunshine Coast: 27 Apr–1 May). We placed nests approximately 800 m apart along the shore to minimize the possibility of the same predator depredating more than one nest sequentially, whilst still achieving adequate replication and dispersion within each region. We aimed for equal replication levels at each site, but due to logistical constraints (travel times, access) the number of experimental units varied between 27 and 38, with a total of 132 nests for the entire study (Table 2).

**Table 2:** Summary of fates after five days of experimental clutches on ocean-exposed beaches and dunes at four study sites in southeast Queensland, Australia, during Jan–May 2015.

Fate of Nest	Moreton Island		Noosa North Shore		Bribie Island		Sunshine Coast		Total	
Carnivores										
Ghost crab			1	*(3%)*					1	*(1%)*
Torresian crow	10	*(27%)*	1	*(3%)*	12	*(44%)*	1	*(3%)*	24	*(18%)*
Red fox			2	*(5%)*			4	*(13%)*	6	*(5%)*
Snake			1	*(3%)*					1	*(1%)*
Carnivores all species	10	*(27%)*	5	*(13%)*	12	*44%*	5	*(17%)*	32	*(24%)*
Domestic dog							1	*(3%)*	1	*(1%)*
Flooded	17	*(46%)*	1	*(3%)*	1	*(4%)*	16	*(53%)*	35	*(27%)*
Intact	7	*(19%)*	27	*(71%)*	4	*(15%)*	7	*(23%)*	45	*(34%)*
People			5	*(13%)*	2	*(7%)*			7	*(5%)*
Sand burial	2	*(5%)*			7	*(26%)*			9	*(7%)*
Unknown	1	*(3%)*			1	*(4%)*	1	*(3%)*	3	*(2%)*
	37		38		27		30		132	

We positioned model nests to mimic natural plover nesting habitat (Lomas et al., 2014), using the following criteria: (i) position on the beach: on the upper part of the unvegetated beach above the spring tide drift line near the base of the dunes (narrow strip of accumulated wrack deposited by swash), in the fore-dune area (the dune slope above the lowest vegetation line up to the first crest), or in the dune area (landward of the top of the fore-dune); (ii) microhabitat exposure: under vegetative cover, in the open next to flotsam, or in the open at least 2 m away from flotsam; (iii) microhabitat topography: in a hollow/swale/depression, or on a mound/ridge. We randomly assigned nests to each nest location category using a randomisation function in Microsoft Excel, which was reapplied until we achieved ‘reasonable’ balance of nests between factor levels to enable subsequent analysis. We determined the placement of the clutch perpendicular to the water’s edge by walking to the midpoint of the dune or fore-dune at predetermined GPS coordinates and selecting the nearest location that met the randomly allocated microhabitat criteria. For beach nests, we used the upper beach just below the foredune to most closely mimic the location of real plover nests (Lomas et al., 2014; Maslo, Handel & Pover, 2011). To allow equal chances for predation from both diurnal and nocturnal predators, we deployed half the nests in the early morning and the other half just after sunset. There was no significant difference (*p* = 0.79) in depredation between nests deployed near sunrise (24%) and those deployed near sunset (26%).

We then monitored nests for a 5-day period with a digital passive infrared (PIR) motion sensor camera (Scoutguard SG560Z-8M) concealed within 1–2 m of each nest, checking daily for signs of predation. Camera operations followed protocols developed for beach scavengers (Huijbers et al., 2015; Lomas et al., 2014). All work was conducted under Animal Ethics Permit No. AN/A/14/84 issued by the University of the Sunshine Coast, and the Scientific Purposes Permits WITK14608214 and WISP14609114 issued by the Queensland Government Department of Environment and Heritage Protection.

### Environmental variables

We measured a suite of microhabitat attributes for each experimental nest. We recorded vegetation characteristics as three complementary variables: (i) small-scale (1-m^2^ quadrat) plant cover (%) centred at the scrape (digital photograph and Coral Point Count (CPCe) software); (ii) height and distance of vegetation nearest to nest (measuring tape); and (iii) dune-wide vegetation cover (line intercept along transects extending from the base of the foredune to the landward edge of the feasible (potential) plover nesting habitat. We used a theodolite to measure four complementary metrics that described the local geomorphology: (i) distance and elevation of the nest relative to the storm drift-line (visible as an accumulation of wrack on the upper beach near the dunes); (ii) distance and elevation relative to the seaward base of the foredune (defined as a distinct rise in the angle of the beach-face); (iii) ‘exposure’ defined as the elevation of the nest relative to the nearest two profile survey points along the beach-dune transect; and (iv) dune dimensions (max. dune ridge height, width of the dune field). We obtained distances of nests from the nearest creek, rocky headland, and house using Google Earth. To index the type and intensity of human activity, we counted (once every day during nest and camera checks) the number of campsites, swimmers, fishers, and dog walkers during approximately 10 min within 100 m of each experimental nest. Data collection occurred between 0700–1100 hours consistently across all deployments. To account for potential differences in human use of shores with respect to weekends, every site was sampled at least once during the weekend and four times during the week.

**Figure 2:**
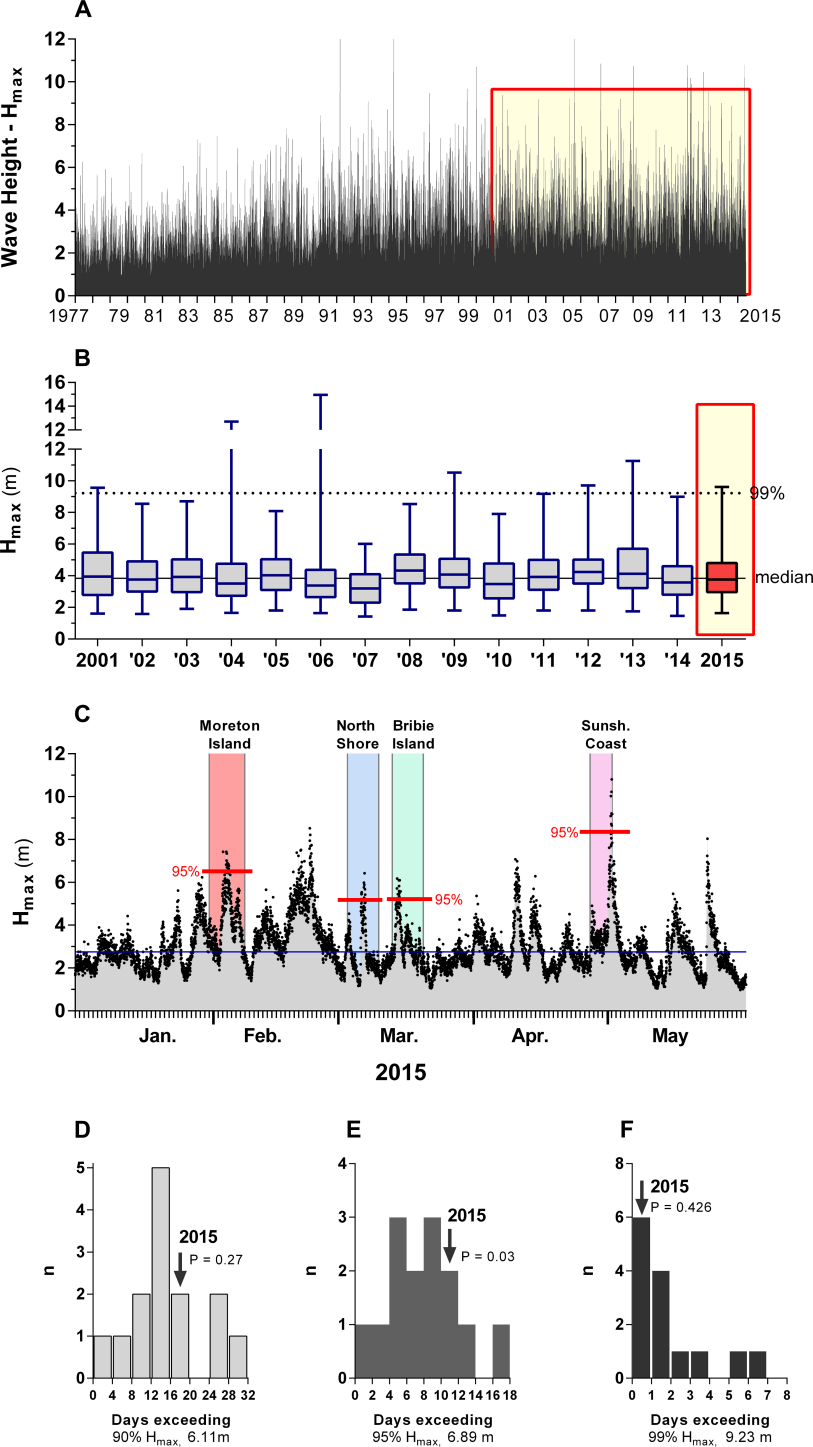
Wave height data recorded offshore (ca. 10 km) from the beaches on which experiments in nest survival of shore- and dune-nesting plovers were conducted in early 2015 (cf. Fig. 1 for region locations), SE-Queensland, Australia. (A) –longer-term record of maximum wave height from 1977 to 2015. (B) –Summary of maximum wave height for the first five months of each year from 2001 to 2015 for which data at comparable recording frequency (30 min) were available; (C) –maximum wave height during the study; (D) –comparison of the frequency of large wave events between 2015 and the period 2001–2014. Large wave events are defined as days on which maximum wave height exceeded the 90th (D), 95th (E) or 99th (F) percentile of the historical record; *p* values refer to *t*-tests contrasting the 2015 value with the mean of the preceding 14 years.

We gathered wave data from wave-rider buoys operated by the Queensland Government (http://www.qld.gov.au/environment/coasts-waterways/beach/monitoring/), using recordings from the Brisbane buoy because it was close to the sites and contained the longest and most complete time-series for the region. Wave heights differed significantly (ANOVA, *P* < 0.005) among sites (Fig. 2).

### Data analysis

To identify the most important predictors of clutch fate, we first built a conditional inference tree using the party package in the statistical programming environment R (Hothorn, Hornik & Zeileis, 2006; R Core Team, 2014). This routine works by testing the hypothesis that the response variable clutch fate, discrete with five levels: intact; flooded; depredated; disturbed by people; or other (covered by sand, or unknown) is independent of the predictors. If this hypothesis is rejected, the routine selects the predictor that has the strongest association with the response and splits the data in two so as to best represent this association. The routine is then repeated recursively on each of the resulting nodes, stopping in each case only when the null hypothesis of no association cannot be rejected. The strength of this approach lies in the fact that because all predictors are reused at each instance of binary partitioning, the routine can reveal not only important predictors, but also statistical interactions and possible nonlinearity.

We supplemented the results of the conditional inference tree with conventional generalized linear modeling (glm) for predated clutches. Clutches lost to flooding were almost perfectly explained by the inference tree, while there were too few observations for disturbance by people to support a more detailed analysis. Our glm employed a logit link function (binomial family) and modeled clutch fate (depredated = 1; intact = 0) as a function of all available unconfounded predictors. In this sense, we considered geographic coordinates, presence of humans (including off-road vehicles and dogs), and both wave and tide metrics confounded with site, because there was no within-region variation for any of these predictors. We used a forward stepwise model-building approach based on the AIC corrected for finite sample sizes (AICc), considering only main effects. This decision to omit interactions was based on initial inspection of the data, which indicated a lack of replication and/or contrast in data across potential interaction cells (especially those associated with site). Following the forward stepwise selection process, we interrogated the final model by recalculating the AICc for all possible combinations of variables used in model building (Quinn & Keough, 2002) and used multi-model inference to determine the relative importance of predictors based on their summed Akaike weights (Burnham, Anderson & Huyvaert, 2011; Symonds & Moussalli, 2011). Finally, we used standard log-likelihood ratio tests to simplify the model by dropping predictors one at a time, starting with the least important, until only significant terms (*α* = 0.05) remained.

## Results

### Causes of model clutch loss and spatial variability

Flooding and depredation were the main causes of clutch failure, accounting for 27% and 25% of all nests lost, respectively. Of the 132 nests deployed, 45 (34%) survived the full 5-day experimental exposure period, 32 were depredated, 35 were lost due to flooding, and seven were destroyed by people (Table 3). Crows were the main predator, accounting for 75% of all depredated clutches; red foxes accounted for 19% of depredated nests, whilst a single clutch each was eaten by a ghost crab and a snake (Table 3). Red foxes visited a further eight nests at the Noosa North Shore and Sunshine Coast, but they did not consume or detect the clutches in these instances. A domestic dog trampled one clutch, and nine clutches were buried by wind-blown sand.

**Figure 3:**
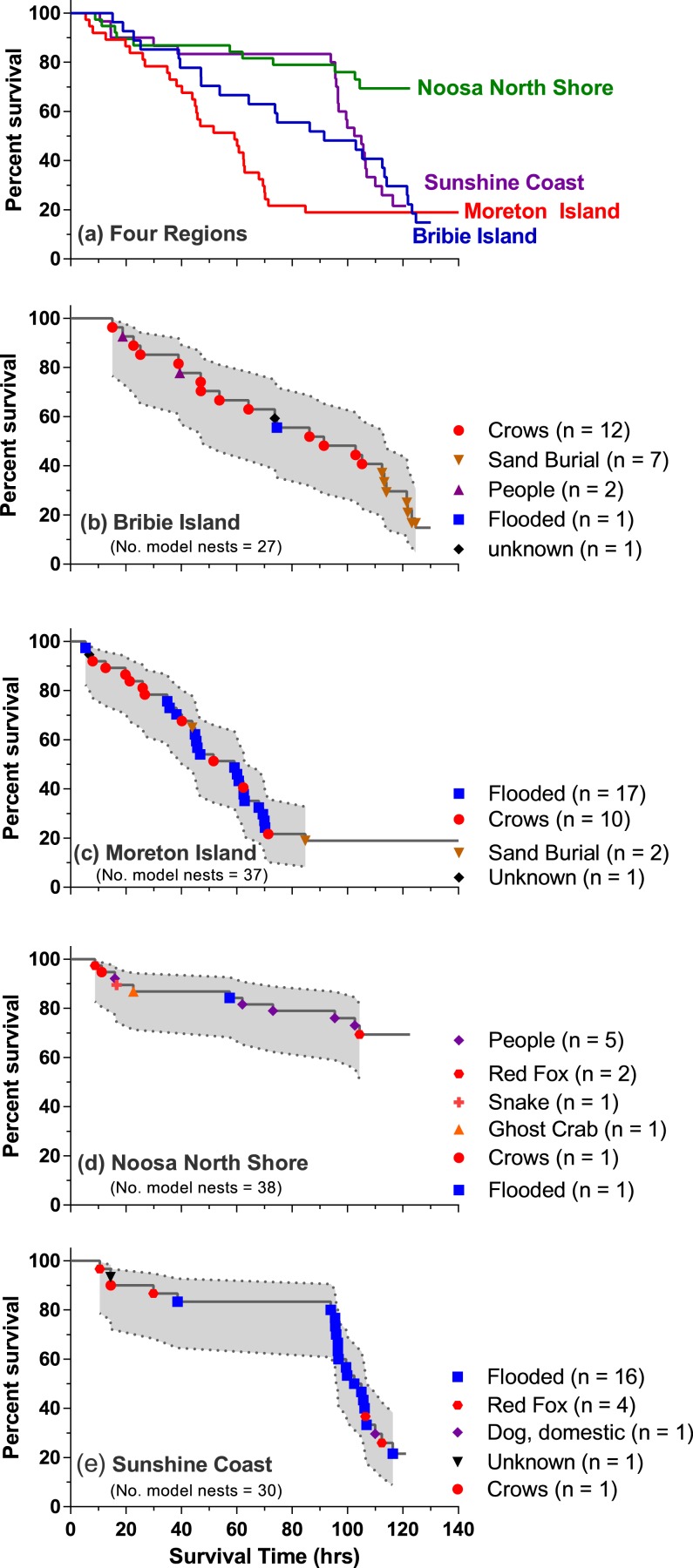
Survival curves for: (A) all sites (Moreton Island, Bribie Island, Noosa North Shore, and the Sunshine Coast, Queensland, Australia) and (B–E) individual sites showing identified causes of nest loss over the time span of the experiments in each region (numbers in parentheses are the actual number of experimental clutches lost attributed to a specific cause; shaded areas are 95% confidence intervals of survival).

Overall clutch survival differed significantly among sites (Mantel Cox test, Chi square = 20.26, *df* 3, *P* ≤ 0.001; Fig. 3). Clutches placed on the Noosa North Shore survived longest and in highest numbers compared with all other sites (Mantel – Cox min. *P* = 0.039; min. Hazard Ratio –North Shore: Other Site = 0.40, 95% CI [0.19–0.72]). Survival patterns were comparable for nests placed at Bribie Island and the Sunshine Coast (Mantel – Cox *P* = 0.96; Hazard Ratio –Bribie Island: Sunshine Coast = 0.98, 95% CI [0.53–1.81]). Clutches on Moreton Island had significantly lower survival rates than those on the Sunshine Coast (*P* = 0.02, Hazard Ratio –Moreton: Sunshine Coast = 1.97; 95% CI [1.12–3.45]) but were comparable to those on Bribie Island (*P* = 0.27; Hazard Ratio - Moreton: Bribie Island. = 1.37, 95% CI [0.79–2.37]).

**Table 3:** Summary of final binomial generalized linear model used to predict the probability of depredation for experimental nests placed in the four study regions within SE-Queensland, Australia, 2015. Estimates for model coefficients refer to log-odds ratios and are additive on the model intercept, which represents the log-odds of depredation at Bribie Island.

	Estimate	Std Err	z	*p*-value
(Intercept)	2.964	1.018	2.912	0.0036
Region Moreton Island	0.078	0.9738	0.081	0.9358
Region Noosa North Shore	−3.660	0.906	−4.041	5.33×10^−05^
Region Sunshine Coast	−0.604	0.976	−0.619	0.5360
Dune Height (m)	−1.106	0.432	−2.561	0.0104
Dist. to Vegetation (m)	0.011	0.005	2.095	0.0362

**Notes.**

Null deviance: 99.099 on 73 degrees of freedom.

Residual deviance: 68.765 on 68 degrees of freedom.

AIC: 80.765.

**Figure 4:**
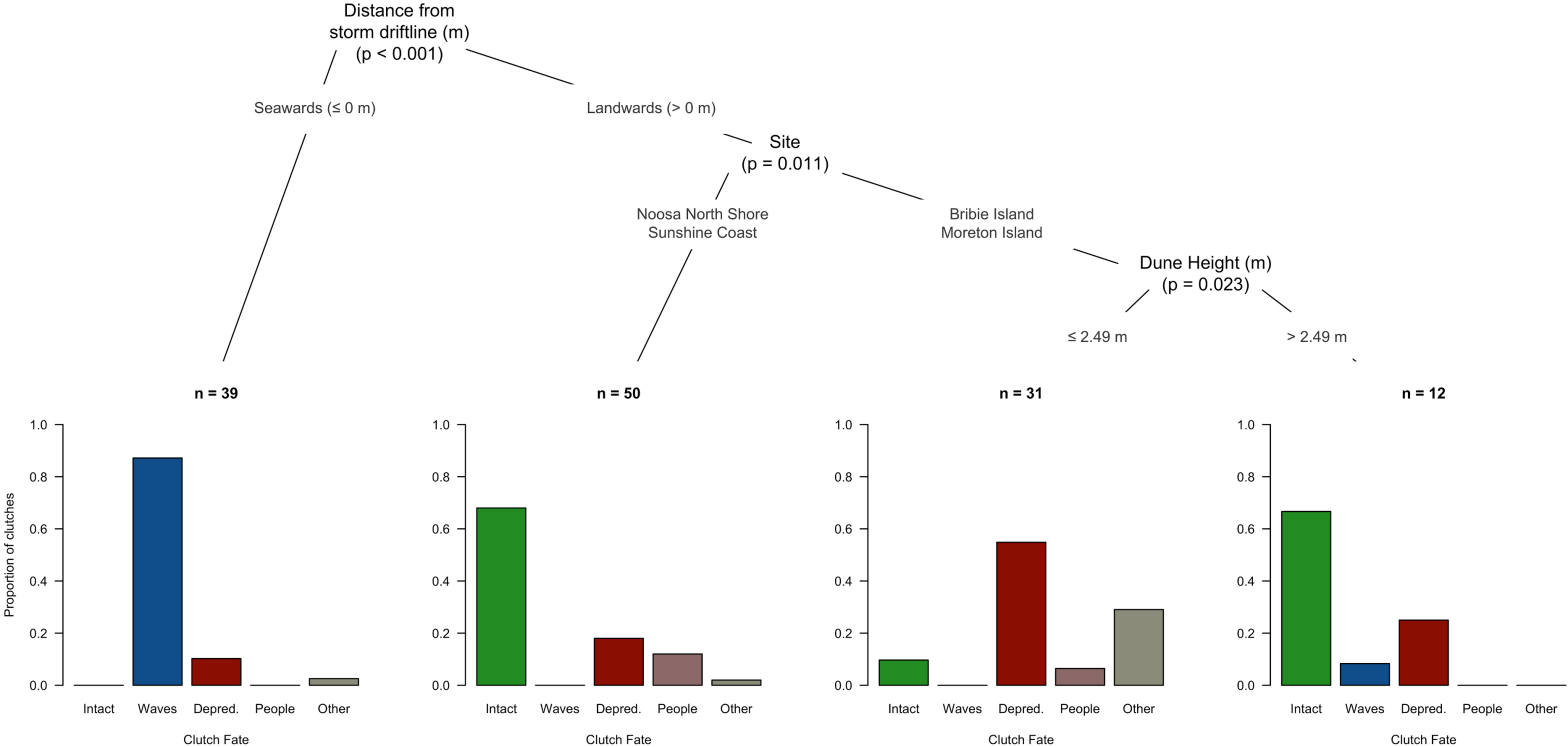
Conditional inference tree indicating the primary predictors of each clutch fate category for all experimental nests placed with the study region of SE-Queensland, Australia, 2015: intact, flooded, depredated, disturbed by people, and other (buried by sand, unknown). Variables are ranked based upon the strength of their association with specific clutch fates and their quantitative values are split to best represent the relevant association.

The conditional inference tree (Fig. 4) strongly resolved distance from the drift line as a significant (*P* < 0.001) predictor of flooding. Thirty-four of the 35 flooded nests in the study were located on the upper beach above the spring. Site was a strong predictor of fate of the remaining 97 clutches (*P* = 0.011). At mainland sites (Noosa North Shore and Sunshine Coast), most of the 50 clutches not situated low on the shore remained intact (*n* = 34) for the duration of the study, with the remainder depredated (*n* = 9), disturbed by people (*n* = 6), or lost to other causes (*n* = 1). The group of nests from Bribie and Moreton Islands that was not lost to flooding was further split on the basis of dune height (*P* = 0.023). Of the 31 nests located where dunes were smaller than 2.49 m in height, more than half were destroyed by predators (*n* = 17). By contrast, where dunes were taller than 4.5 m, only 3 of the 12 nests were lost to predators (Fig. 4).

The forward stepwise binomial glm identified site, distance to nearest creek, dune height and distance to nearest vegetation as the most important predictors (in order of addition to the additive model) of depredation. Subsequent multi-model inference provided a slightly different prioritization (relative importance of predictors based on their summed Akaike weights in parentheses): site (1.00); dune height (0.77); distance to nearest vegetation (0.72); and distance to nearest creek (0.69). Log-likelihood ratio tests indicated that removing distance to nearest creek from the model did not cause a significant deterioration in model fit (ΔDeviance = 2.369, Δ*DF* = 1, *p* = 0.124), but that the subsequent removal of distance to nearest vegetation did cause the fit to deteriorate significantly (ΔDeviance = 4.634, Δ*DF* = 1, *p* = 0.031). The final predictive model therefore included main effects for region, dune height, and distance to nearest vegetation, and explained 31.3% of the null deviation.

**Figure 5:**
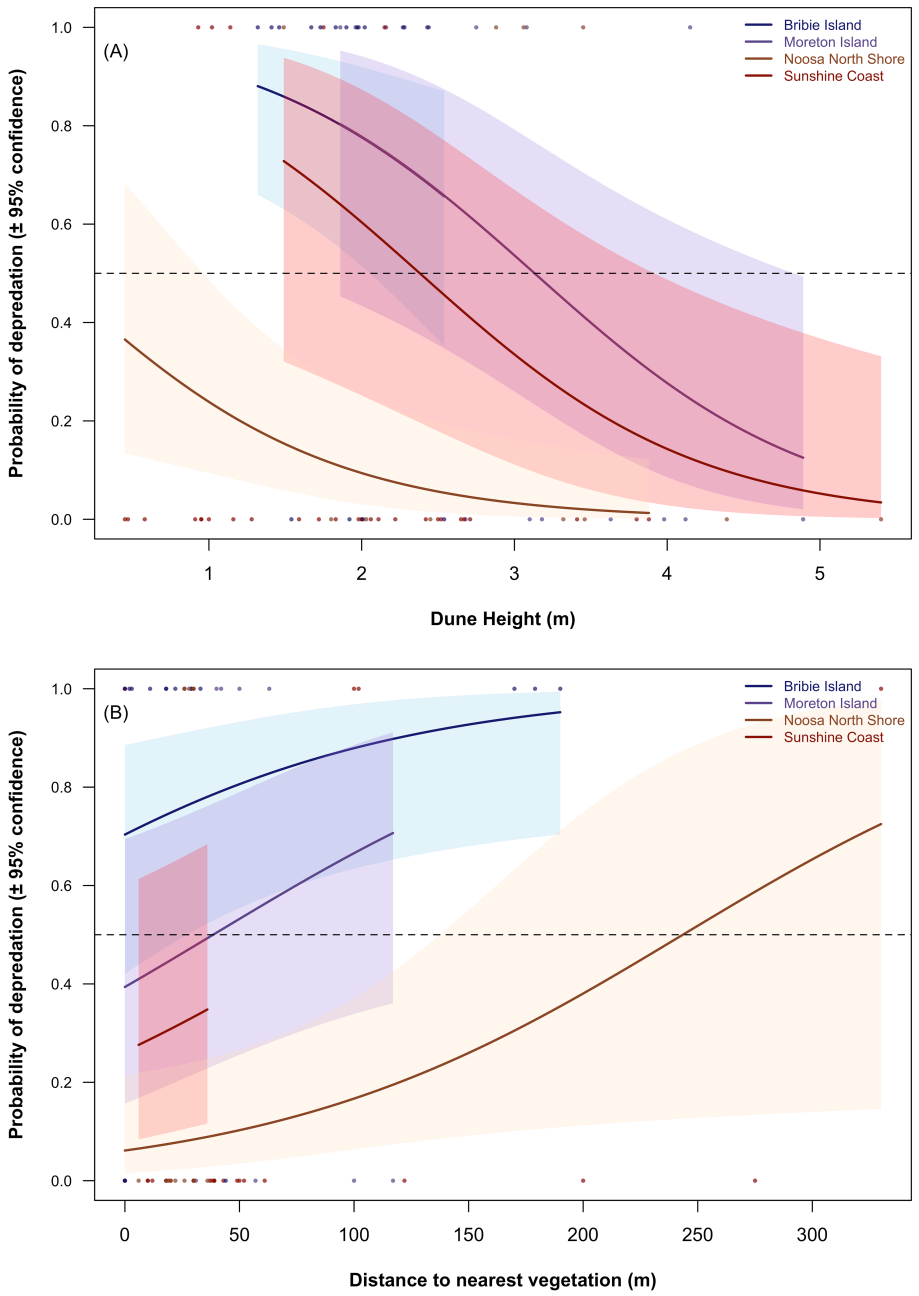
Influence of dune height and distance to nearest vegetation on the probability of experimental clutch depredation across all study sites within SE-Queensland, Australia, 2015. Shaded regions indicate 95% confidence intervals.

Coefficients from the final model indicate that at average values for dune height and distance to nearest vegetation for each site, probability of depredation was significantly higher than would be expected by chance at Bribie Island, significantly lower than would be expected by chance at Noosa North Shore, but no different from null expectation at either Moreton Island or the Sunshine Coast. The odds of depredation declined (*P* = 0.010) by a factor of 0.33 for every metre of dune height and increased (*p* = 0.036) by a factor of 1.01 for every additional metre away from the nearest vegetation (Table 3, Fig. 5).

## Discussion

Our broad-scale evaluation of the relative importance of multiple drivers of clutch loss on beaches suggests that predation and flooding are important causes of clutch failure in a regional context. Contrary to expectations that predation pressure is particularly strong on human-dominated beaches (Seneviratne et al., 2012), predation across the entire study region was high, even at sites with no or little human development. Specifically, corvids were responsible for the majority of predated nests, complementing the suite of local-scale studies that increasingly demonstrate corvids as the primary cause of clutch loss in beach- and ground-nesting birds (Ekanayake et al., 2015b; Madden, Arroyo & Amar, 2015). As human commensals, corvids have rapidly expanded their global population, with the highest densities occurring in areas of significant urbanization (Hardy & Colwell, 2012; Lauro & Tanacredi, 2002; Rees et al., 2015b.). Corvids are highly mobile, moving readily between natural and highly urbanized areas (Whisson, Weston & Shannon, 2015), and their impact on reproductive success can be extreme (Burrell & Colwell, 2012; Lima, 2009). While corvid densities are typically higher in urbanized areas (Marzluff et al., 2001; Rees et al., 2015a), the spillover effect of increased corvid recruitment has cascading effects on the avian species in periurban environments (Marzluff et al., 2001). A troubling aspect of intense corvid depredation is the limited and hitherto unconfirmed effectiveness of management responses available. Very few studies examine the effects of corvid management on breeding bird productivity, and they report small or equivocal effects (Forys et al., 2015; Neatherlin & Marzluff, 2004; Velasco, 2015).

Our results indicate that predation risk is higher for nests occurring in low-lying dunes away from vegetation, and the biological explanation for this phenomenon remains unclear. Corvids typically land within 1 m of the nest and approach on foot, or they discover nests while walking around open substrate (Forys et al., 2015; Velasco, 2015). Therefore, it is possible that corvids, as visual predators (Ekanayake et al., 2015b), are less able to detect or access nests in thicker vegetation (Rees et al., 2015b.). Nest placement in shorebirds is partly about survival of the clutch, but also about survival of the parents (Gillis et al., 2012; Tieleman, Noordwijk & Williams, 2008); early detection of incoming predators enables adults to survive to re-clutch multiple times within a season and/or breed over several or many subsequent seasons (Dowling & Weston, 1999; Lomas et al., 2014). There may also be sublethal stress effects of incubating nests away from cover (Amat & Masero, 2004).

The sizeable proportion of clutches that failed due to flooding emphasized the severity of flooding as a threat to coastal ground-nesting birds (Pol et al., 2010; Van De Pol et al., 2010). Although many of the flooded nests in this study occurred during stormy weather on the Sunshine Coast (53%) and Moreton Island (45%), we found little evidence that the type of large wave occurrences that led to clutch losses in 2015 were atypical events. The number of days in which maximum wave height exceeded the 90th percentile of historical records for the first five months of each year (*n* = 17 days) was not significantly different from the mean of 14.6 days in the previous 14 years, with only four years (2001, 2008, 2009, 2013) exceeding values recorded in 2015 (Fig. 2). Similarly, wave heights exceeded the top 1% of the historical record on only a single day during the present study. The impact of storm or high tide events can be catastrophic to bird reproduction, particularly if storms occur late in the incubation period when there is little time for re-nesting. There exists considerable uncertainty about when storms will arise, where they will be most severe, and for how long habitats will be impacted (Hemer et al., 2013; IPCC, 2013; Schlacher et al., 2015b), suggesting that assessment of flood risk for beach-nesting birds is imperfect (Lomas et al., 2014). Human-mediated flood risk abatement, if feasible, may be of great importance to population viability.

Human disturbance accounted for the loss of ≤9 clutches at any site and only 11% across the entire region, illustrating the relatively weak influence of this source of failure on beach-nesting bird reproductive success. Human presence in the vicinity of a nest was not related to the probability of clutch loss to direct human causes, as would be expected. In fact, we found no significant effect of dogs, camping, off-road vehicles or proximity to development on the probability of a nest being disturbed. These results suggest that passive management of human disturbance (i.e., symbolic fencing, signage) is effective in reducing anthropogenic impacts to beach-nesting birds in a regional context. Where human disturbance of nesting birds is severe in a specific location, localized management can address the problem (Del Viejo et al., 2004; Ruhlen et al., 2003; Weston & Elgar, 2007).

## Caveats

The results described here result from the placement of model clutches within suitable plover habitat. Model clutches are used extensively in research and when implemented carefully can reveal important ecological patterns and processes (Berry & Lill, 2003). We acknowledge beach-nesting birds theoretically select nesting sites to reduce the risks of clutch loss, perhaps basing their choice in part on previous nesting experiences. We also recognise that model clutches are not associated with incubator behaviour, which either can be protective (i.e., defence) or may render clutches more vulnerable to predation (e.g., visual and scent cues) (Ekanayake et al., 2015a). Indeed, model clutch studies are enlightening in terms of physical destruction of the eggs (e.g., Buick & Paton, 1989), but they shed no light on the impacts of disturbance (disruption of incubation which may reduce egg viability), even though disturbance to breeding shorebirds is considered a conservation threat (Maslo, Burger & Handel, 2012; Meager, Schlacher & Nielsen, 2012; Powell & Collier, 2000; Quinn et al., 1996; Schlacher, Nielsen & Weston, 2013; Weston, Schlacher & Lynn, 2014). In a review of 80 studies, (Major & Kendal, 1996) report that artificial nests underestimate actual survival of real nests, while cameras on nests either do not affect or slightly increase clutch survival (Richardson, Gardali & Jenkins, 2009; Sanders & Maloney, 2002). Ekanayake et al. (2015a) and Ekanayake et al. (2015b) also used model clutches on a study of red-capped plovers in southern Victoria and confirmed the comparability of natural and model clutches in identifying egg predators.

Model nests also permit otherwise infeasible studies to be performed. While common, widespread nesting species may enable the study of real nests in some areas to address some research questions (none were available on coasts in subtropical Australia). However, this would represent a biased research effort because studies would be confined to species apparently coping well with prevailing conditions; traditional yet abandoned habitats could not be assessed for threats. Artificial nests permit an examination of egg predation risk in areas of suitable but often unoccupied habitat, as we have done here. Calibration of this model egg study with real clutch fate and survival (impossible here because no such data, which is strictly comparable, are available) would not only be confirmatory, but would shed light on the utility of studies that use model eggs to guide real nest management. We also note that survival estimates of real clutches are often biased (e.g., Nichols et al., 1984).

## Management Implications

As coastal bird populations continue to decline globally (e.g., Rodrigues et al., 2004), the enhancement of reproductive success through active management plays an increasingly critical role in species’ persistence. Our analysis suggests that management of human disturbance through symbolic fencing, signage, and regulatory measures across a region is likely enough to effectively mitigate anthropogenic impacts. The significant influence of predation and flooding on clutch loss at the regional scale demonstrates that actively addressing these threats will lead to increased viability of beach-nesting bird populations. Predator management is typically performed at the site scale and can be effective in the short-term, and targeted application may increase clutch success (e.g., exclosures, electric fencing, removal; Maslo & Lockwood, 2009; Neuman et al., 2004). However, predator mortality may elicit a compensatory response (i.e., increased reproduction) in affected populations and new individuals can quickly occupy open niche space (Harding, Doak & Albertson, 2001; Smith et al., 2010). Both corvids and foxes can disperse great distances from their natal territories (Dekker, Stein & Heitkönig, 2001; Marzluff et al., 2001); therefore, targeting source populations within the region may have trickle-down benefits to beach-nesting birds. Predation management, however, may be unnecessary and inefficient if nests are highly vulnerable to flooding.

Managing flood risk of clutches is likely difficult to implement. Possibilities include modifying habitats to maintain nesting areas at higher elevations, for example through dredge spoil or ecologically sensitive beach nourishment. Identification of the sites within a region where habitat modifications have the highest likelihood of providing benefits to the population will be critical. At the location scale, moving nests or raising them before flood events can also be attempted. In flood prone locations, a tractable strategy may be to assess a clutch’s vulnerability to flooding (Haig et al., 2005; Sanders & Maloney, 2002; Thomas, Lanctot & Székely, 2006) and then manage failure risk for the subset of nests that are not flood prone (this also preserves any learnt, adaptive, nest placement whereby birds learn to avoid flood prone habitats).

In short, conservation of coastal bird species in the presence of multiple threats and measureable uncertainty requires practitioners to make hard choices about management intervention. An understanding of the driving forces of clutch success at a regional scale offers managers some confidence in developing strategies that promote the viability of coastal bird populations (Meager, Schlacher & Nielsen, 2012) rather than mitigating impacts on a site-by-site or location-by-location basis.

##  Supplemental Information

10.7717/peerj.2460/supp-1 Data S1 Raw data

## Additional Information and Declarations

### Data Availability

The following information was supplied regarding data availability: Maslo B, Schlacher T, Michael W, Huijbers C, Anderson C, Gilby B, Olds A, Connolly R, Schoeman D Data from: Regional Drivers of Model Clutch Loss Reveal Important Trade-offs for Beach-nesting Birds: http://dx.doi.org/10.5061/dryad.hn132.
